# Embryodysgenesis of the Lids and Orbit

**DOI:** 10.1038/s41433-026-04740-6

**Published:** 2026-07-17

**Authors:** James A. Katowitz, Yasaman Ataei, Elizabeth J. Bhoj, Jesse A. Taylor

**Affiliations:** 1 Department of Ophthalmology, The Perelman School of Medicine, University of Pennsylvania, Philadelphia, PA USA; 2 Division of Ophthalmology, The Children’s Hospital of Philadelphia, Philadelphia, PA USA; 3 Department of Pediatrics, The Perelman School of Medicine, University of Pennsylvania, Philadelphia, PA USA; 4 Division of Human Genetics, The Children’s Hospital of Philadelphia, Philadelphia, PA USA; 5 Division of Plastic Surgery, The Perelman School of Medicine, University of Pennsylvania, Philadelphia, PA USA; 6 Division of Plastic, Reconstructive and Oral Surgery, The Children’s Hospital of Philadelphia, Philadelphia, PA USA

**Keywords:** Paediatrics, Disease genetics

## Abstract

The 2025 Cambridge Ophthalmology Symposium focused on “Embryogenesis and the Eye”. This review discusses key mechanisms in embryodysgenesis contributing to abnormal development of the eyelids and bony orbit. Most of these anomalies can be categorized as **malformations**, primarily divided into two groups: **1. Premature Craniosynostoses** and **2. Congenital Craniofacial Clefting Disorders**. Etiologic factors influencing both categories are reviewed, along with advantages of an adaptation of the **Tessier clockface system** in classifying craniofacial clefts into four regional areas. Current diagnostic and management concepts for these two major congenital craniofacial groups are reviewed.

## Introduction

Embryodysgenesis of the lids and orbit refers to abnormal craniofacial development occurring between the **4th and 8th weeks of gestation**, resulting in a wide spectrum of congenital anomalies [1].

The most common disorders arising during this period are:

1. **Premature Craniosynostoses**
2. **Congenital Craniofacial Clefting Disorders**.

A third group, encompassing a heterogeneous array of rarer malformations, will not be discussed in detail here.

In the craniosynostoses, **premature fusion of cranial sutures and fontanels** restricts brain expansion, resulting in characteristic skull and facial deformities. Growth is normally driven by the expanding brain, which increases tenfold in size between weeks four and eight of gestation, doubles in weight by six months in utero, and triples by one year postnatally [1, 2]. The sutures serve as *passive expansion joints*; premature closure along specific sutures disrupts this process, producing regionally predictable deformities.

**David W. Smith’s dysmorphology classification**, now in its eighth edition, remains particularly useful for describing congenital anomalies as:

- **Malformations** – intrinsically abnormal formation of tissue
- **Deformations** – external mechanical forces on normal tissue
- **Disruptions** – destruction of normal tissue
- **Dysplasias** – disorganized cellular development within tissue

Craniofacial anomalies may originate as single defects, sometimes evolving into *sequences*—where a primary anomaly triggers a cascade of secondary changes. Syndromic cases represent multiple related anomalies with a common genetic basis [3]. Comprehensive evaluation of all children with congenital craniofacial deformities requires a **multidisciplinary team approach**, including genetic consultation, pediatric subspecialty assessment, and psychosocial support for the patient and family.

### Normal craniosynostosis formation

Normal growth of the skull is regulated by a delicate balance of proliferation and differentiation occurring within the sutures [4–7]. The process of craniosynostosis occurs from human neural crest cells migrating from the neural tube to form the calvarial bone complex with differentiation of cells into osteoblasts and chondrocytes via a complicated interplay of multiple pathways.

## Premature craniosynostoses

**Premature suture closure** often occurs from an imbalance in pressure between the expanding brain and any resistance external or within the surrounding cranial bone [7]. Intra-uterine pressure from twins can cause premature synostosis. In addition, teratogenic factors such as anticonvulsants like valproate or ingested drugs such as fentanyl are additional environmental factors. A third mechanism can be due to genetic factors observed in relatives or confirmed by appropriate genetic sequencing tests [8].

The dysmorphology framework classifies craniosynostoses as **malformations** or **deformations**, depending on etiology [3]. Clinically, they are divided into:

- **Syndromic craniosynostoses** – typically genetic, producing more complex craniofacial dysmorphisms such as midface retrusion and exorbitism.
- **Non-syndromic craniosynostoses** – usually isolated, mechanical, or teratogenic in origin.

### Syndromic craniosynostosis

**Fig. 1:**
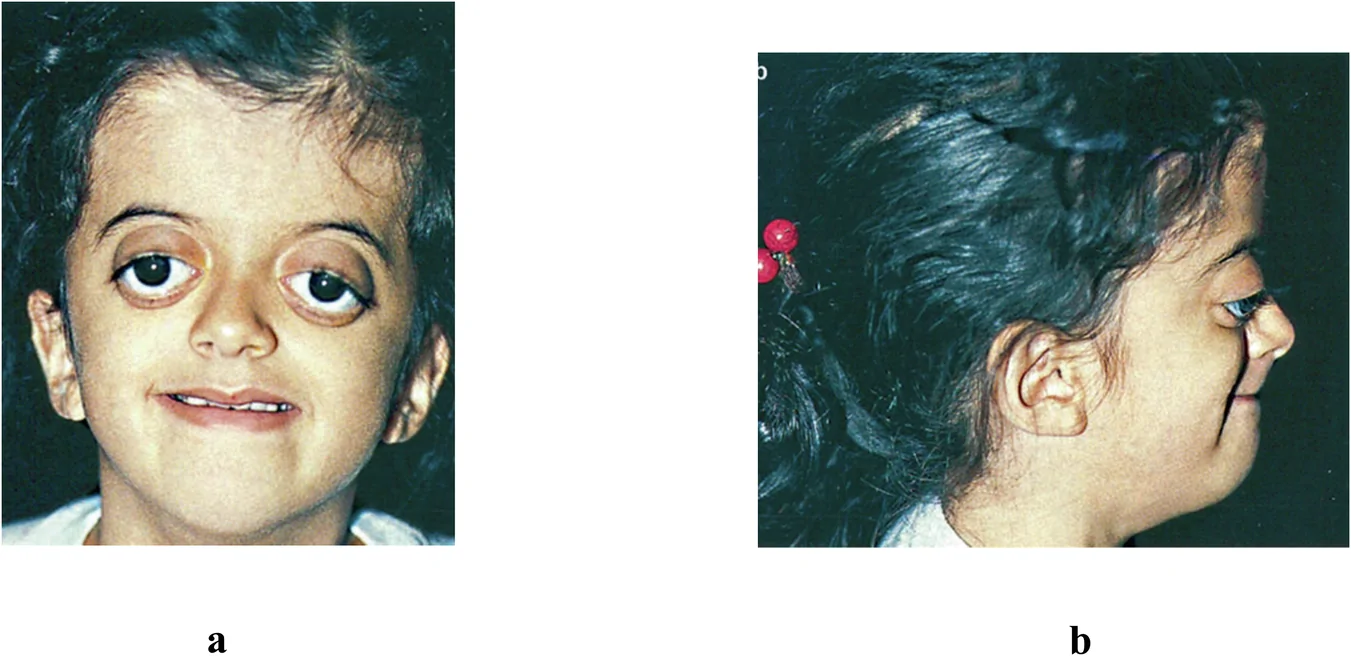
Crouzon Syndrome with exorbitism, corneal exposure, ptosis and lower lid retraction. *Borrowed with permission from Pediatric Oculoplastic Surgery. Second Edition. Springer; 2018. P 895; Fig. 40.23*.

**Fig. 2:**
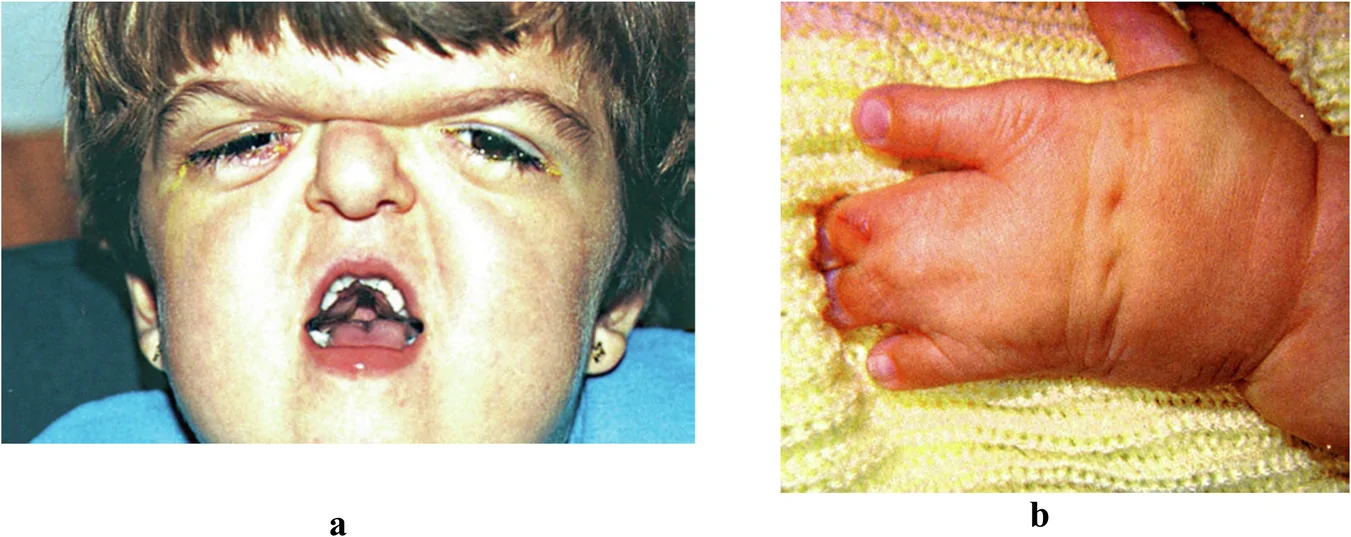
Apert Syndrome. **a** Note midface retraction, beaked nose. **b** Bone and cutaneous syndactaly of 2^nd^, third and 4^th^ digits. *Borrowed with permission from Pediatric Oculoplastic Surgery. Second Edition. Springer; 2018. P 808; Fig. 38.11 & Fig. 38.12*.

Among the syndromic forms, various genetic factors play an important role (Table 1). **Crouzon syndrome** is the most common. It is **autosomal dominant**, often caused by **FGFR2** or **FGFR3** mutations, and occurs in approximately **1.6 per 100,000 live births**. Characteristic findings include midface hypoplasia, exorbitism with corneal exposure, lid retraction, and ptosis, but normal intelligence and extremities **(**Fig. 1**)** **[**9]. **Saethre–Chotzen syndrome**, due to **TWIST1** mutations, presents similarly to Crouzon, but with additional limb anomalies and syndactyly resembling **Apert syndrome** which is particularly distinguished by syndactyly of the hands and feet as well as severe midface retrusion, and craniosynostosis from **FGFR2 gain-of-function mutations (**Fig. 2**)** **[**1]. De novo mutations are frequent and associated with increased paternal age. Incidence is roughly **1 in 100,000 live births (**Fig. 6**)** **[**10–15]. Other syndromic forms include **Pfeiffer,** **Muenke**, and **Carpenter syndromes**, each with overlapping craniofacial phenotypes and varying genetic bases [1, 16–20].

**Table 1:** Categories of Genetic Disorders and Examples.

Disorder Category	Example Disorder	Gene examples	Inheritance	OMIM Number
**Syndromic Craniosynostoses**	Crouzon/Apert	FGFR2, FGRF3	AD	123500
	Saethre-Chotzen	TWIST1	AD	101400
	Other Craniosynostoses	ALPLSKI, CD96, ZIC1, IFT43. RUNX2, WDR19, WDR35, SPECC1L, RECQL4, TCF12, IFT122, MSX2, POR, ERF, FGFR1, Other		
**Non-Syndromic Craniosynostoses**		**Simple form:** most likely deformational intrauterine causes or ingestion of teratogenic agents such as valproate **Persistent form: (about 2%)** have genetic factors such as beta 2 and beta 3 transforming growth factors, BMP2 alleles, FGR and Twist mutations seen in the syndromic forms are rare		

### Non-syndromic craniosynostoses

Non-syndromic forms constitute approximately **85%** of all craniosynostoses. They are more commonly due to **extrinsic mechanical factors** such as intrauterine constraint (e.g., multiple gestation or breech position), though **teratogens** such as **valproate,** **retinoic acid**, and **fentanyl** have also been implicated (Table 1) [21].

**Fig. 3:**
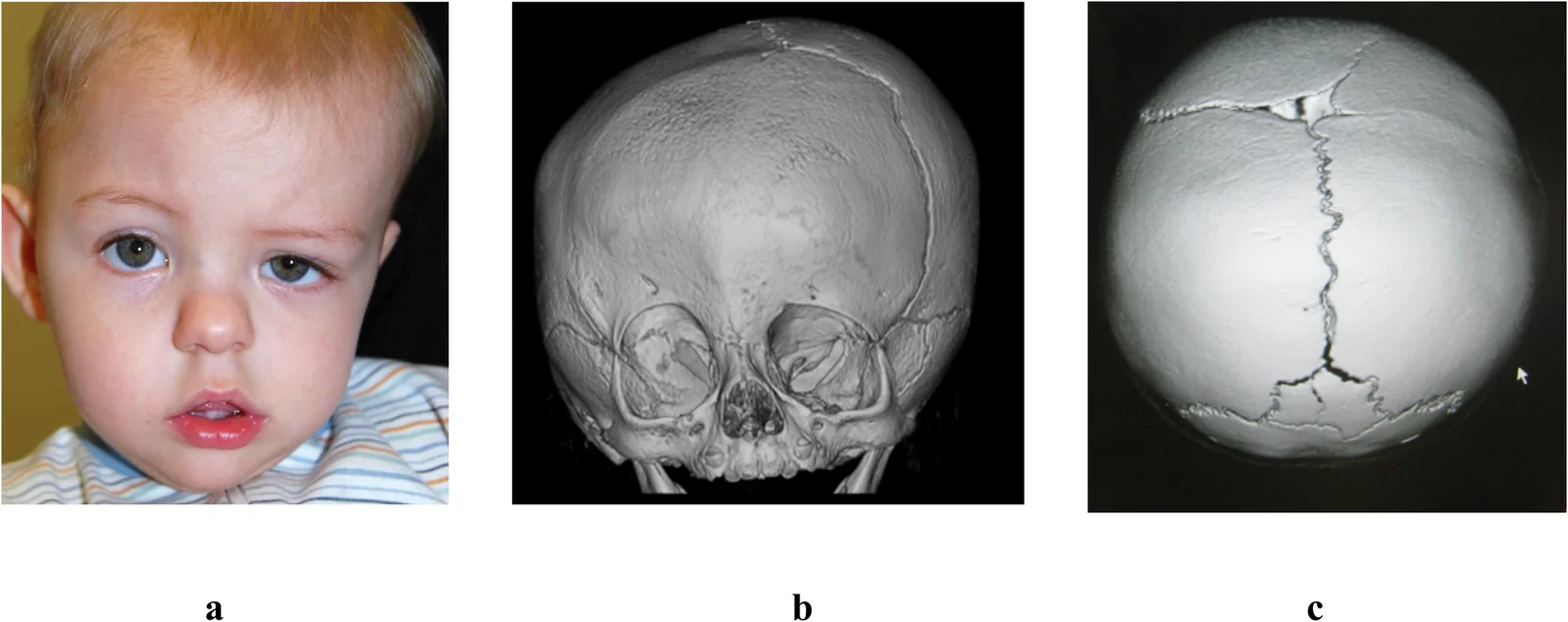
Non-Syndromic craniosynostosis patient with. **a** right superolateral brow elevation and orbital dystopia, **b** CT scan demonstrating retrusion of fright frontal bone and superolateral elevation of the right orbital rim, **c** right coronal suture synostosis. *With permission from Children’s Hospital of Philadelphia (CHOP)*.

Clinically, these cases produce asymmetric craniofacial contours rather than profound dysmorphisms. Typical features include **superolateral brow elevation,** **orbital dystopia**, and **upper orbital rim retrusion (**Fig. 3**)**.

Elevated **intracranial pressure (ICP)** is a critical concern—rising by 14% with a single suture closure and up to **47%** with multiple closures—potentially leading to cognitive and neurodevelopmental impairment if untreated [22]. From an ophthalmologic standpoint, complications include **exposure keratopathy,** **optic pathway anomalies,** **strabismus**, and **refractive errors**. At the Children’s Hospital of Philadelphia (CHOP), over **90%** of patients exhibited strabismus and **80%** had refractive abnormalities [23].

### Surgical management of premature craniosynostoses

Surgical intervention aims to relieve intracranial pressure and restore cranial symmetry.

**Fig. 4:**
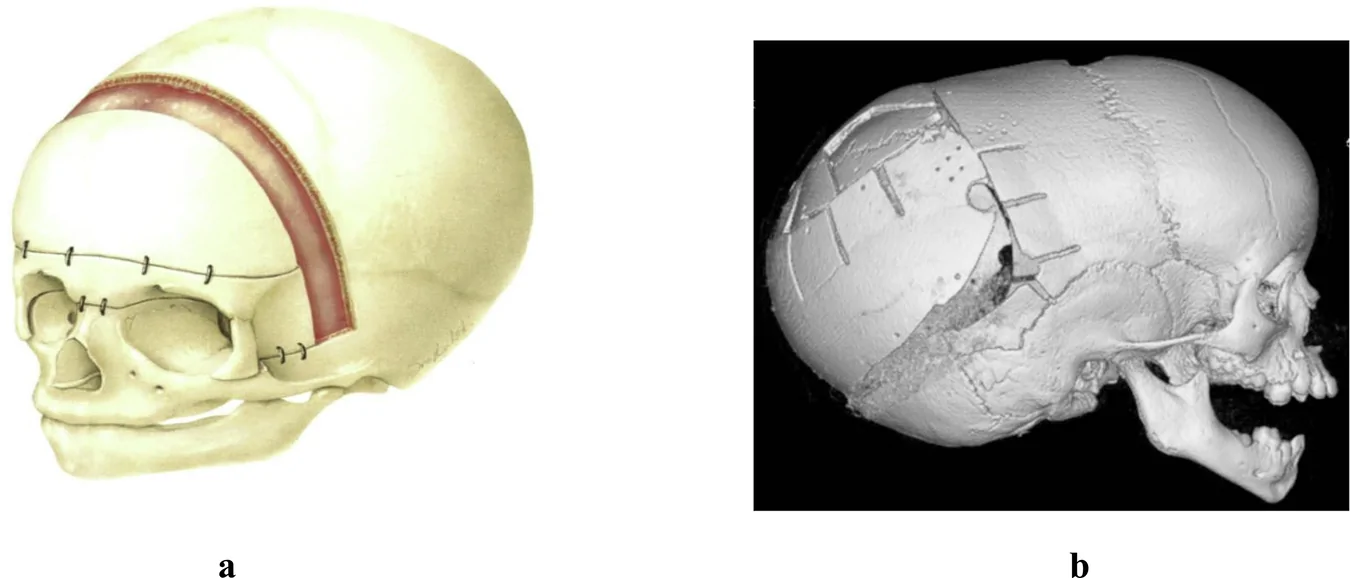
Syndromic Craniosynostosis Repair. **a** strip craniotomy to reduce elevated intracranial pressure, **b** posterior cranial decompression to also adjust calvarial shape. *Borrowed with permission from Pediatric Oculoplastic Surgery. Second Edition. Springer; 2018. p. 895, 884; Fig. 40.24 & Fig. 40.8*.
- **Posterior cranial vault expansion** to permit the brain to grow in an appropriate directions and to correct any associated calvarial deformity can be done by 2–6 months of age **(**Fig. 4b**)** [9]
- **Sagittal craniosynostosis** repair demonstrates the effectiveness of **posterior cranial vault expansion (**Fig. 5**)**

- **Syndromic cases** often require **strip craniectomy** within 2–4 weeks postnatally to accommodate brain growth **(**Fig. 4a**)** **[**9]

**Fig. 5:**
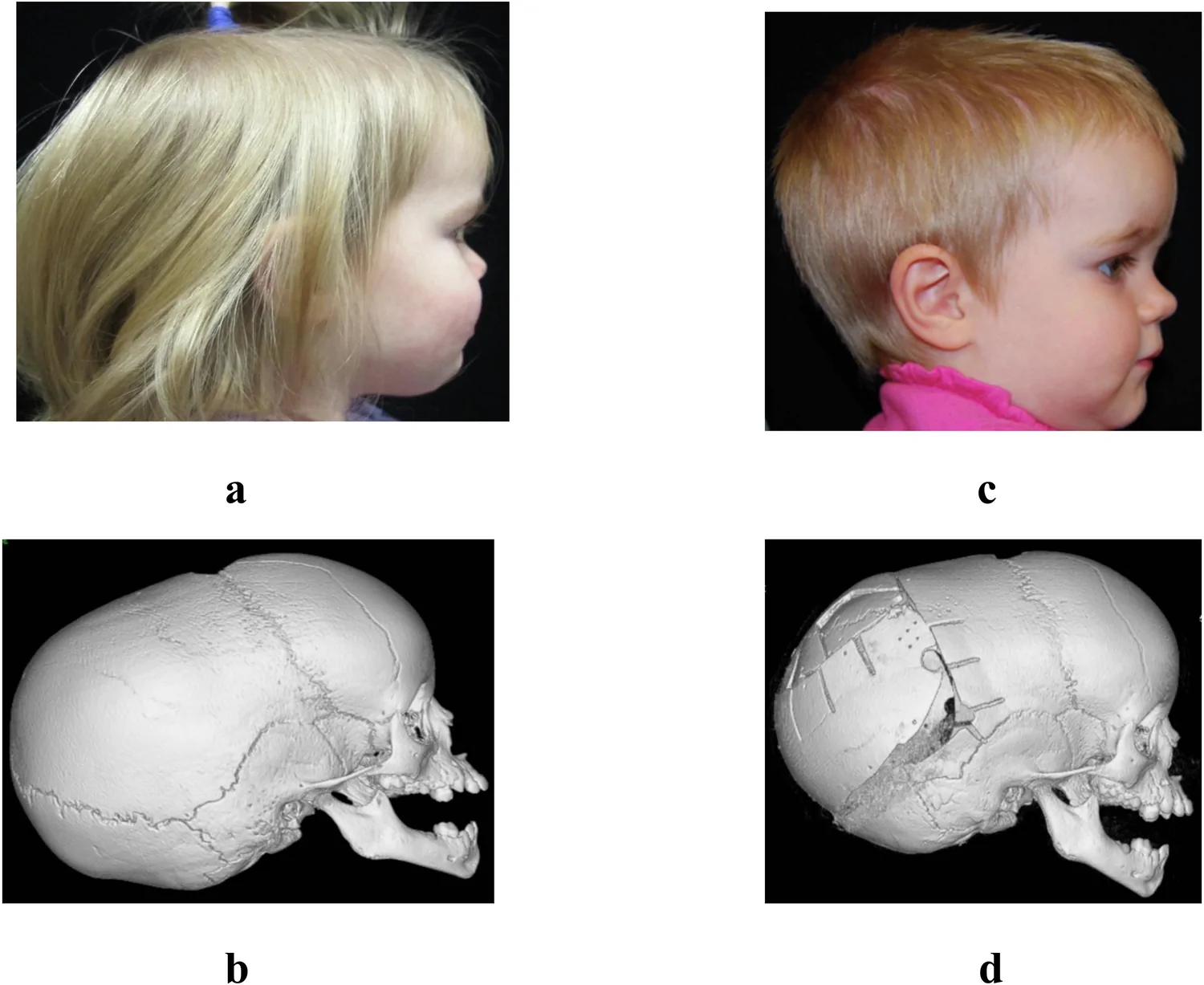
Sagittal Synostosis Repair. **a, b** preop before posterior cranial vault reshaping, **c, d** postop with calvarial length reduced and vertical height increased. *With permission from Children’s Hospital of Philadelphia (CHOP)*.
- **Unilateral coronal synostosis** is typically treated with a **strip craniectomy** and orbital repositioning to correct dystopia **(**Fig. 6**)**

**Fig. 6:**
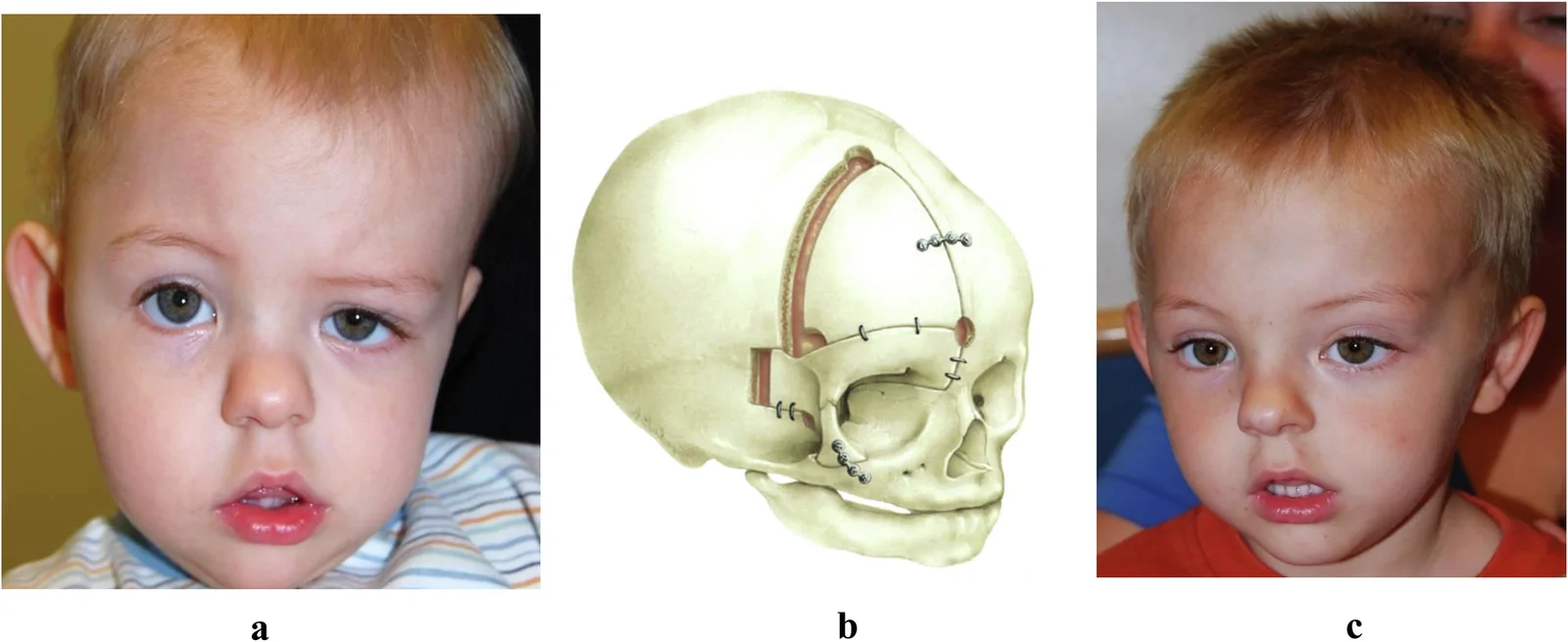
Non-Syndromic Craniosynostosis Repair. **a** Preop with superolateral elevation of the orbit and brow, **b** right orbital advancement procedure, **c** postop with brows and orbits now symmetrical. *With permission from Children’s Hospital of Philadelphia (CHOP)*.
- **Midface advancement** is generally deferred until adolescence due to relapse risk. In severe cases, early staged **Le Fort-type osteotomies** or **distraction osteogenesis** are options, the latter offering controlled bone elongation with reduced morbidity.
- **Endoscopic suture release**, an advancement developed at CHOP, is now yielding shorter anesthesia times, lower transfusion rates, and improved scar outcomes [24].

A recent CHOP review identified **myogenic ptosis** in approximately **10%** of patients with premature craniosynostosis, most commonly associated with **coronal suture** involvement and independent of syndromic status [25].

## Congenital craniofacial clefting disorders

Craniofacial clefts constitute the second major group of embryodysgenetic anomalies affecting the lids and orbit. These malformations occur between **weeks 4 and 8 of gestation**, corresponding to the critical period of fusion among the **frontonasal, maxillary, and mandibular processes** **[**26].

### Etiology of congenital craniofacial cleft formation

**Fig. 7:**
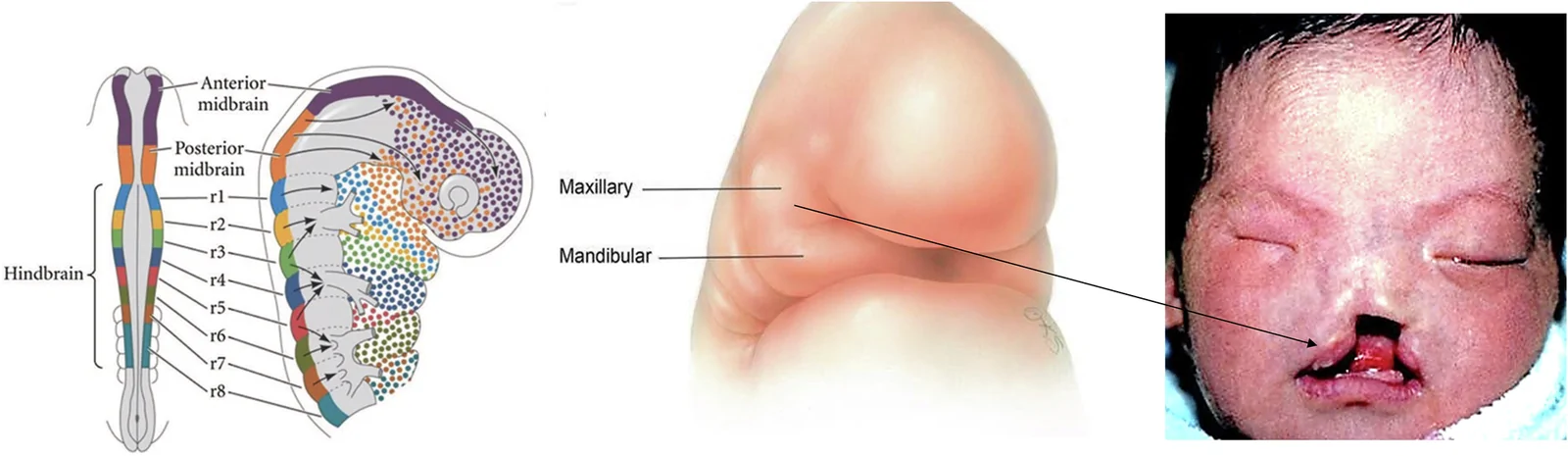
Facial cleft lip formation from failure of neural crest cells to fill the mandibular processes. *Borrowed with permission from Pediatric Oculoplastic Surgery. Second Edition. Springer; 2018. p. 19, 20, 811; Fig. 38.15, Fig. 2.2a, Fig. 2.4b*.

The primary cause appears to be failure of neural crest cell migration resulting in deficient mesenchymal support of the lid folds or specific facial processes with subsequent epithelial collapse **(**Fig. 7) (13**)** **[**9, 22, 23, 27, 28].

Environmental and teratogenic factors (radiation, infection, metabolic disturbance, or exposure to retinoic acid, valproate, or fentanyl) can also disrupt normal morphogenesis. Amniotic bands following amniocentesis can produce **amniotic band–related clefts** (Fig. 14) [1, 28]. Except in **monogenic syndromes** (e.g., *Treacher Collins*, *Stickler*), hereditary transmission is generally limited.

#### A. Tessier clockface system

**Fig. 8:**
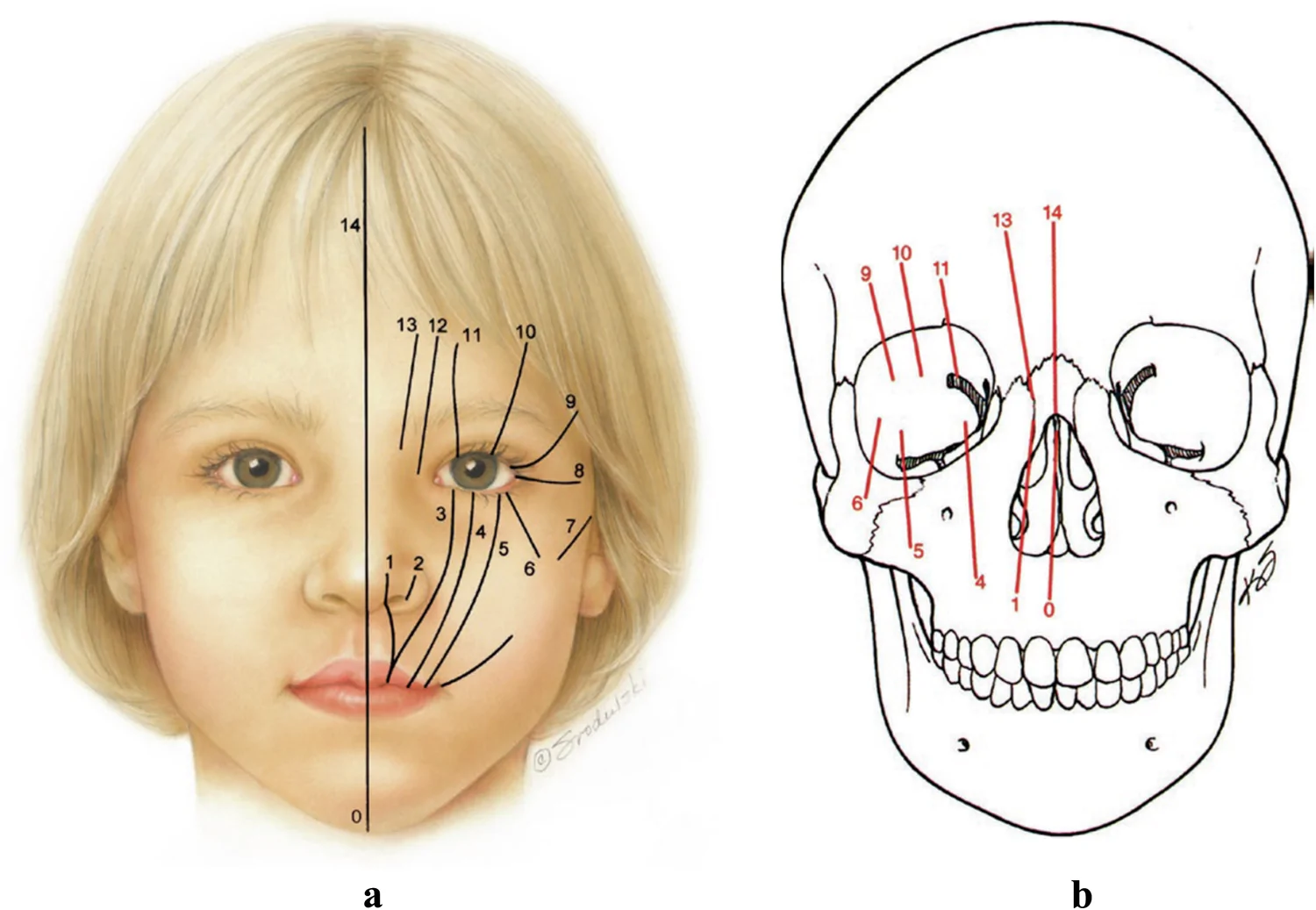
Tessier Clockface System. **a** Soft-tissue clefts, **b** bony clefts. *Borrowed with permission from Pediatric Oculoplastic Surgery. Second Edition. Springer; 2018. p. 812; Fig. 38.16*.

**Fig. 9:**
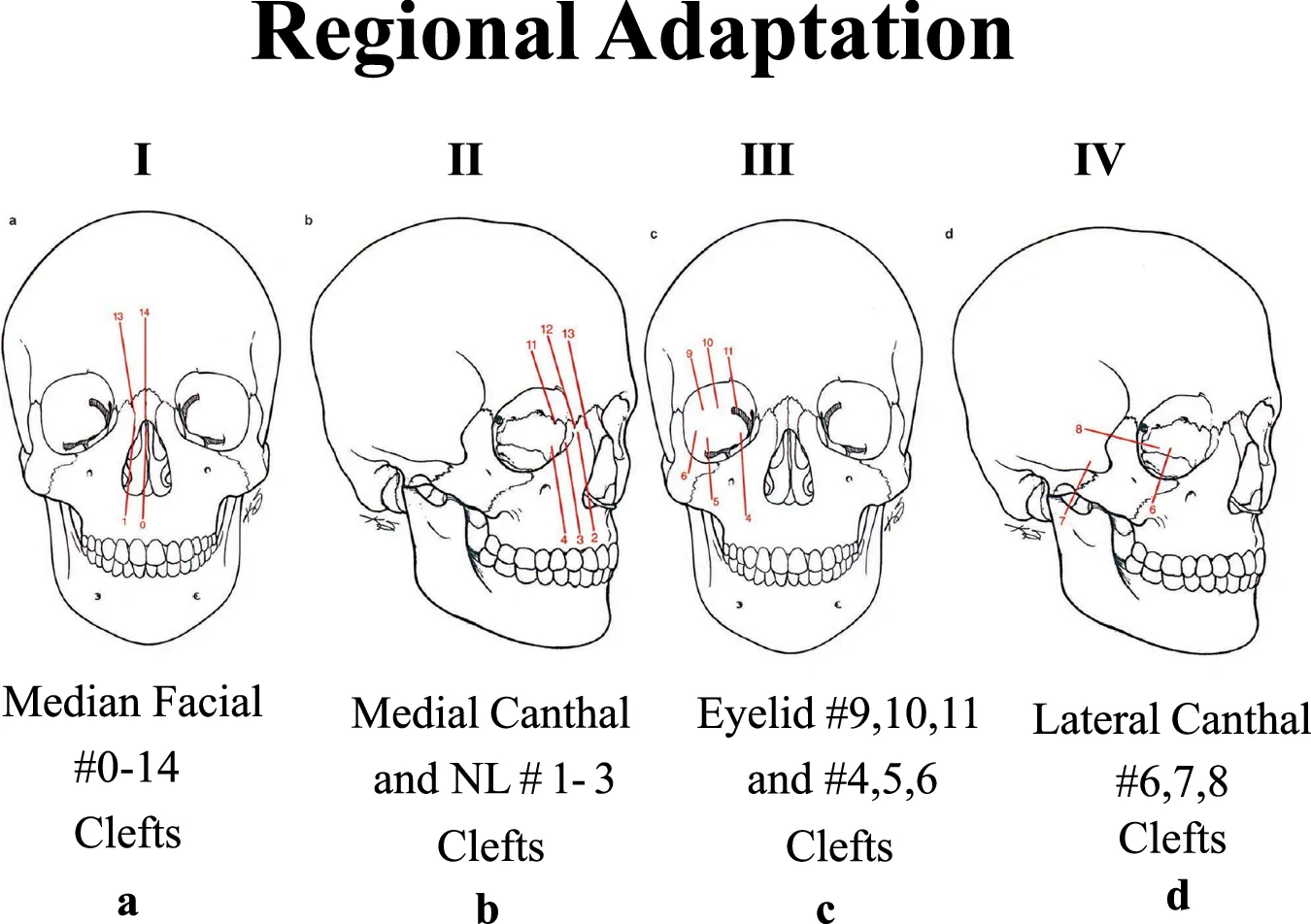
Regional Adaptation of Tessier-based Clockface System with clinical examples in Table 2 and Figs 10–15. *Borrowed with permission from Pediatric Oculoplastic Surgery. Second Edition. Springer; 2018. p. 813; Fig. 38.17*.

The **Tessier clockface classification** provides an anatomic topographic system encompassing both soft tissue and skeletal involvement (Fig. 8). The facial midline corresponds to #0 inferiorly and #14 superiorly; clefts are numbered circumferentially around the orbit **(**Fig. 8**)** **[**1]. For surgical planning, we have found it practical to categorize these clefts into **four regional zones**, progressing from medial to lateral **(**Fig. 9**) (**Table 2**)** [1].

1. **Area I** – Median (#0–14) defects, including encephaloceles.
2. **Area II** – Medial canthal and nasolacrimal clefts (#0–3).
3. **Area III** – Central eyelid colobomas (#9–11).
4. **Area IV** – Lateral canthal and zygomatic clefts (#6,7, 8), include a syndromic triad of *Treacher Collins*, *Goldenhar*, and *hemifacial microsomia*.

**Table 2:** Regional Adaptation.

Region	Presentation	Tessier clefts
**Area I** Median facial clefts	Hypertelorism Encephaloceles	0, 1, 13, 14
**Area II** Medial canthal and nasolacrimal clefts	Median canthal dystopia Nasolacrimal disorders	2, 3,4 11, 12, 13
**Area III** Central eyelid clefts	Colobomatous defects	4, 5, 6 lower 9, 10, 1 l upper
**Area IV** Lateral canthal clefts	Lateral canthal dystopia OVA triad	6, 7,8

**Fig. 10:**
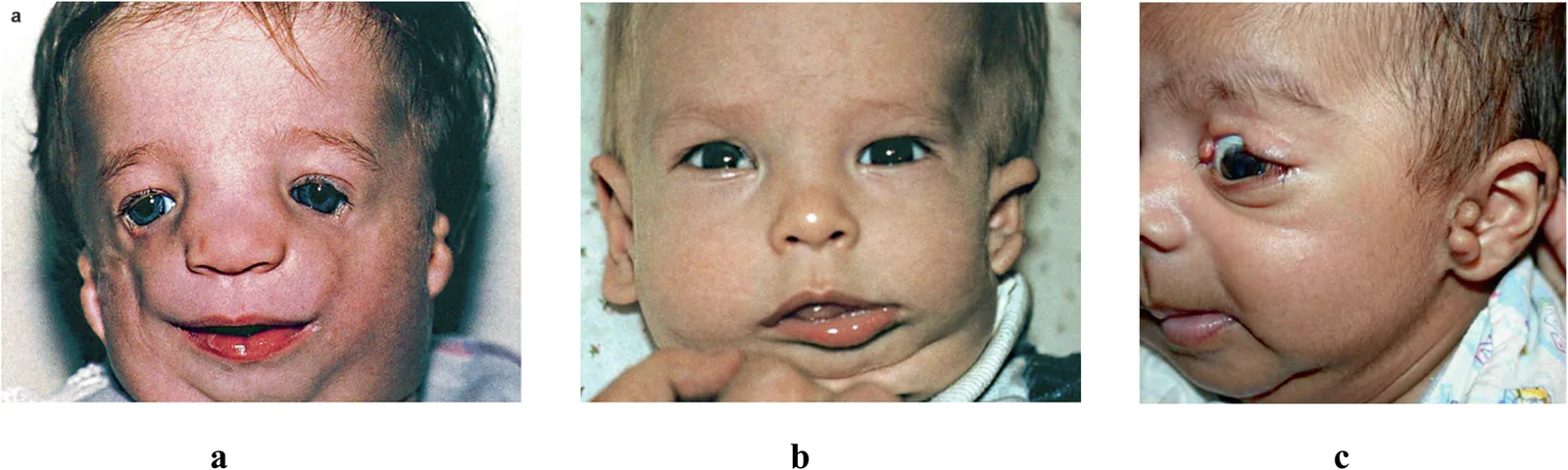
OVA Clinical Spectrum #6,7,8 Triad in Regional Area IV. **a** Treacher Collin Syndrome with symmetrical facial anomalies of lateral canthal dystopias and malar and soft tissue hypoplasia, **b** hemifacial microsomia with unilateral facial soft tissue hypoplasia also involving the bony mandible, **c** Goldenhar Syndrome with upper lid coloboma, lipodermoid under lateral upper lid and ear anomalies. *Borrowed with permission from Pediatric Oculoplastic Surgery. Second Edition. Springer; 2018. p.885, 817, 818; Fig. 40.9, Fig. 38.25, Fig. 38.27*.

**Oculo-Auricular-Vertebral Triad in Area IV (**Fig. 10**)** **[**1, 9]**:**

Gorlin and colleagues described an oculo-auriculo-vertebral spectrum (OAV) consisting of epibulbar dermoids, preauricular appendages, and mandibular hypoplasia. The OAV spectrum can present with a high degree of phenotypic variation. The mildest form, **hemifacial microsomia**, is characterized by unilateral microtia and/or pre-auricular skin tags, At the other end of the spectrum is **Goldenhar syndrome** which can be considered a more severe expression involving microtia with mandibular hypoplasia, cervical spine anomalies, and/or epibulbar dermoids. Teratogenic associations (valproate, fentanyl, etc.) are well documented with this syndrome [29]. **Treacher Collins syndrome** (mandibulofacial dysostosis), the third member of the OAV spectrum, is the one major genetic entity of the OAV triad. It presents as an autosomal dominant anomaly caused by **TCOF1,** **POLR1C**, or **POLR1D** mutations with bilateral zygomatic and mandibular hypoplasia, lateral canthal dystopia and ear anomalies **(**Table 3**)**.

**Table 3:** Categories of Genetic Disorders and Examples.

Disorder Category	Example Disorder	Gene examples	Inheritance	OMIM Number
***Clefting Disorders***				
**Syndromic Facial Clefts**	Treacher Collins (Mandibulofacial dysostosis)	TCOF, POLR1C, POLR1D	AD	154500
	Goldenhar Syndrome	2% possible genetic etiology	AD?	
**Non-syndromic Facial Cleft**	Lid Colobomas Facial microsomias Amniotic bands	**Metabolic etiology:** gestational diabetes **Teratogenic etiology:** retinoic acid, fentanyl and valproate **Mechanical etiology**		

### Surgical management of craniofacial clefts

**Fig. 11:**
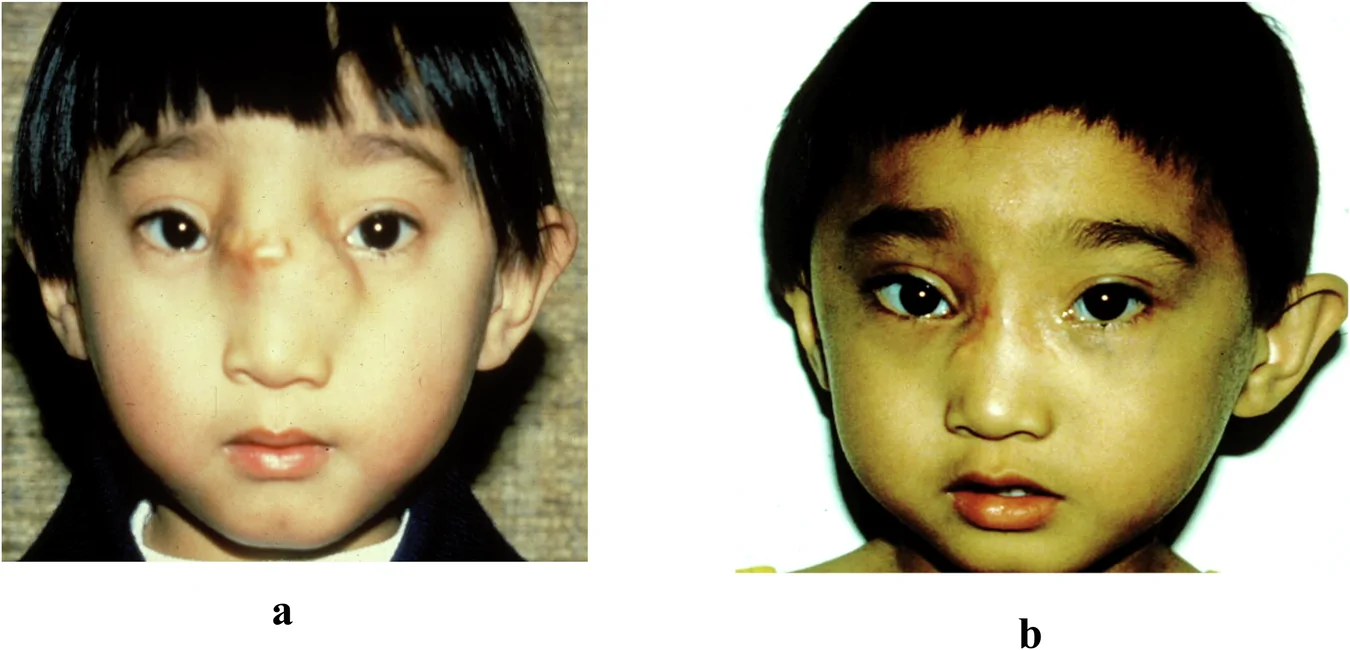
Surgical Repair Area I Tessier #0-1 Midline Cleft with Encephalocele. **a** preop glial excision and bone graft, **b** patient at 6 months postop. *With permission from Children’s Hospital of Philadelphia (CHOP)*.
- **Area II** (medial clefts): medial canthal dystopias and clefts involving the nasolacrimal system often require a combined team effort. **(**Fig.12**)** **[**1, 28]

Applying the **Regional Tessier-based system** facilitates procedural planning:

- **Area I** (median clefts/encephaloceles): Managed with excision of glial tissue and cranial bone grafting. **(**Fig. 11**)** **[**1]

**Fig. 12:**
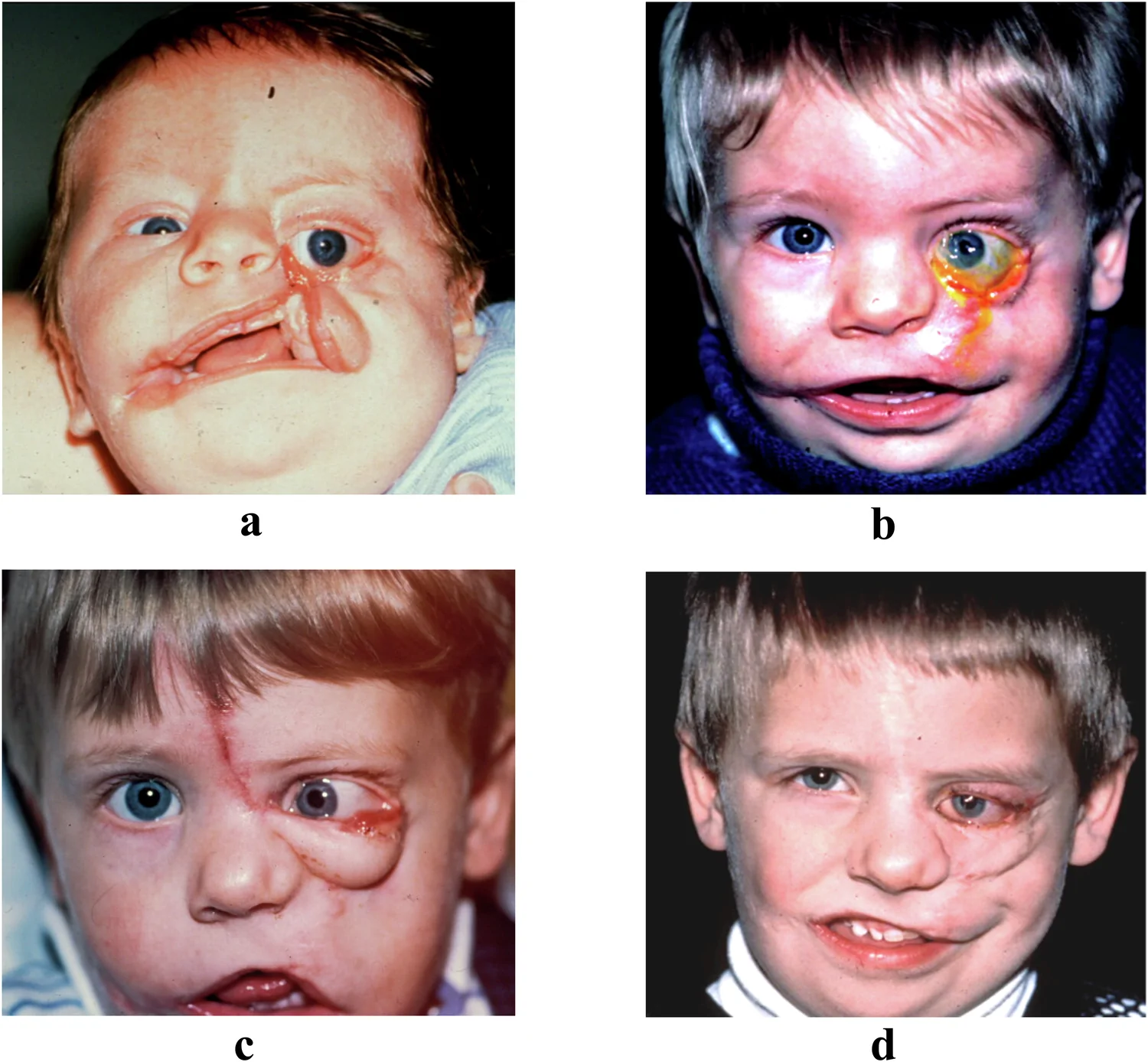
Surgical Repair Area II Tessier #4 cleft. **a** Defect caused by pressure from a swallowed amniotic band, **b** priority for feeding mandated cleft lip repair first resulting in lower lid retraction with corneal exposure controlled with topical lubrication, **c** mid-forehead flap utilized to reconstruct lower lid, **d** postop debulking and lateral canthoplasty. *Borrowed with permission from Pediatric Oculoplastic Surgery. Second Edition. Springer; 2018. p. 814; Fig. 38.20 and with permission from Children’s Hospital of Philadelphia (CHOP)*.
- **Area III** (central lid colobomas) **(**Fig. 13**)** [28]
  -  < 50% upper lid defects: direct repair with canthotomy/cantholysis and levator advancement to prevent secondary ptosis **(**Fig. 14**)** **[**28]
  - >50% upper lid defects: require staged lower lid-sharing procedures, delaying the second stage several weeks to establish vascularity and then severing the flap to avoid amblyopia
  - Complex cases such as clefts involving multiple areas often require a staged multidisciplinary approach [28].

**Fig. 13:**
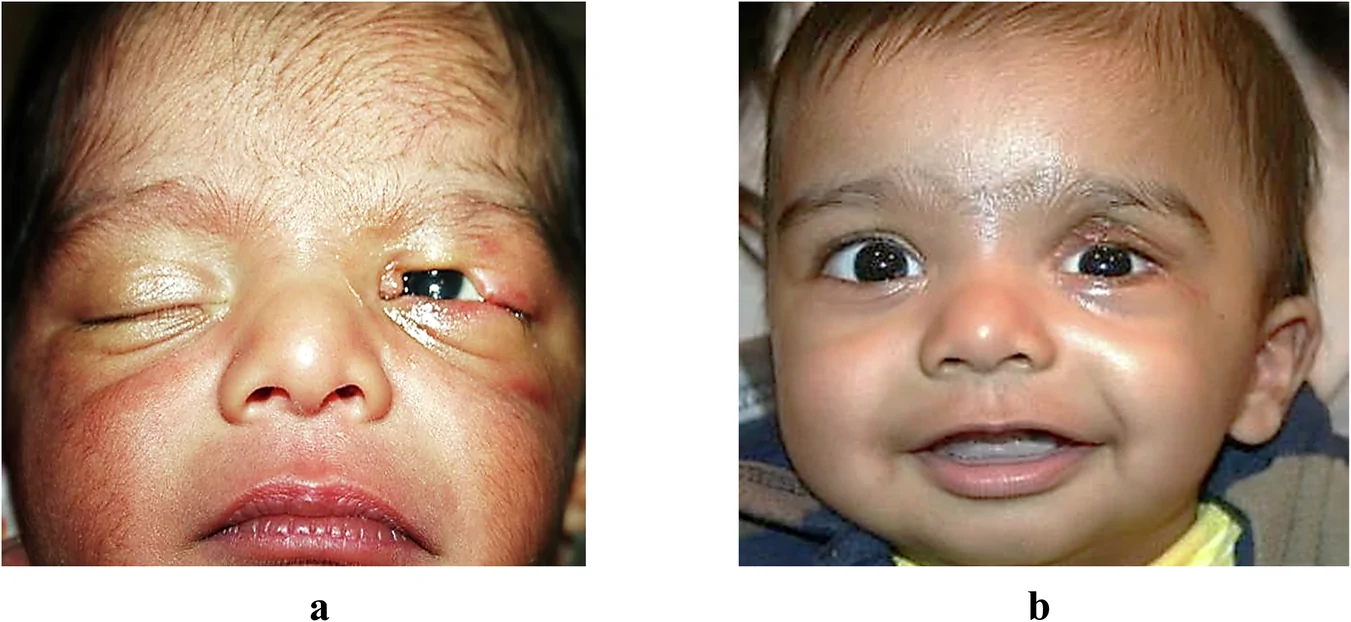
Surgical Repair Area III # 9,10,11 Central Coloboma Clefts: < 50%. **a** Horizontal repair can be done for smaller defects, the technique is similar to a lid laceration repair with extension of the lid crease to permit a levator muscle advancement, **b** 3-months postop coloboma repair with levator muscle advancement; note good lid level and contour avoiding a secondary ptosis repair. *Borrowed with permission from Pediatric Oculoplastic Surgery. Second Edition. Springer; 2018. p. 323, 324; Fig. 21.13 and Fig. 21.14*.
- **Area IV** (lateral canthal/zygomatic clefts): Mild cases can be corrected by transposition flaps; Severe cases may require bone grafts, fillers, or even microvascular free flaps for soft-tissue restoration **(**Figs. 14, 15**)** **[**28, 30].

**Fig. 14:**
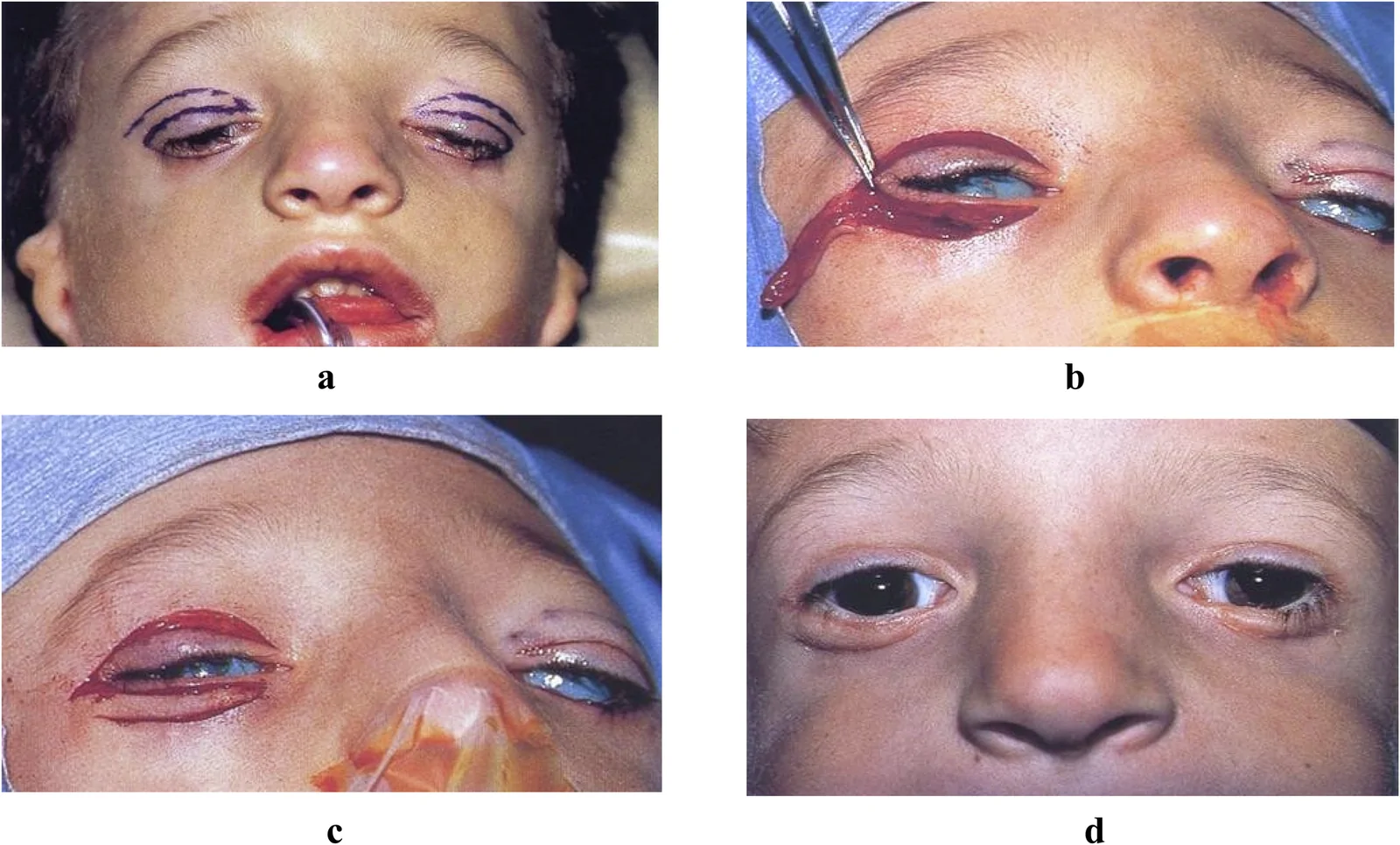
Surgical Repair Area IV # 6,7,8 Clefts. **a** Preparation of bilateral flaps for mild Treacher Collins Syndrome: a. marking transposition, **b** dissection of skin/muscle flaps, **c** transposition of flaps into lower lids prior to bilateral canthopexies, **d** patient at 1 year postop. *Borrowed with permission from Pediatric Oculoplastic Surgery. Second Edition. Springer; 2018. p. 330; Fig. 21.20*.

**Fig. 15:**
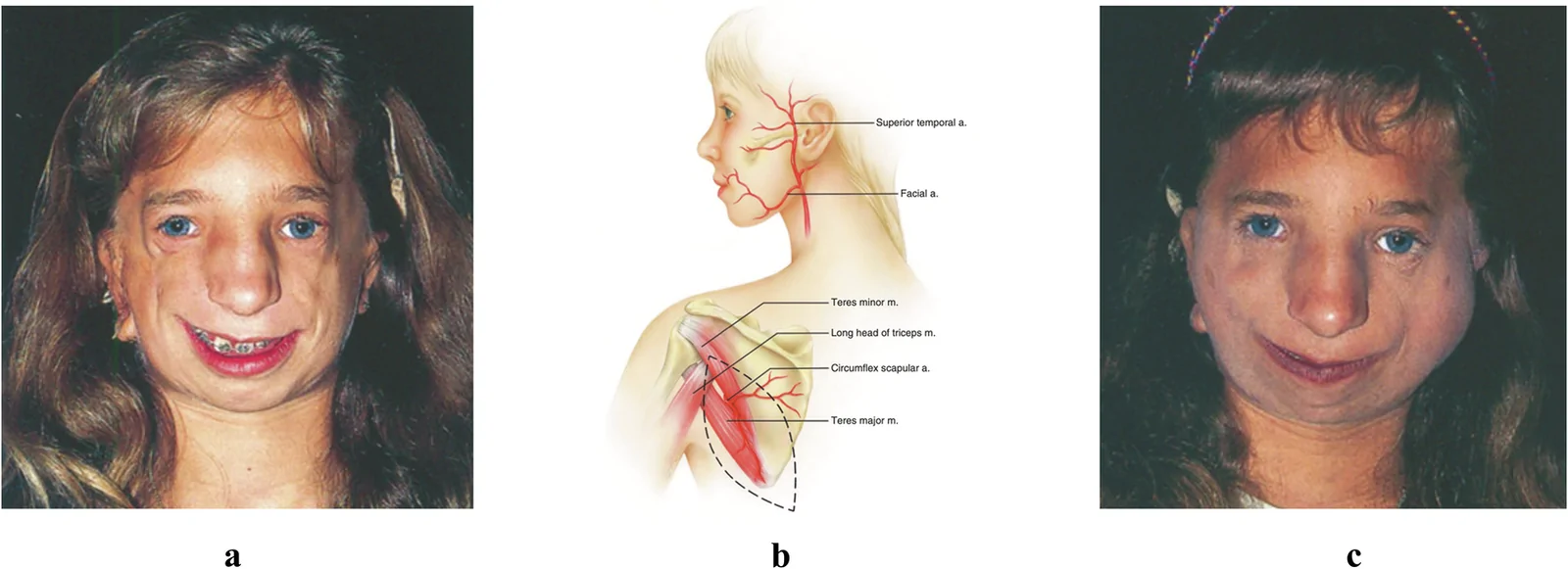
Complex Surgical Repair Area IV #6,7,8 Clefts. **a** An 11-year-old female with Treacher Collins syndrome prior to bilateral facial augmentation with a scapular facial free flap, **b** Illustration of scapular flap anatomy, including two common facial arterial recipient sites, **c** Four months post bilateral scapular flap augmentation. *Borrowed with permission from Pediatric Oculoplastic Surgery. Second Edition. Springer; 2018. p. 161, 162; Fig. 8.23 and Fig. 8.24a,c*.

## Other craniofacial malformations

Less common congenital anomalies include **anophthalmia,** **microphthalmia,** **cryptophthalmos**, and **blepharophimosis–ptosis–epicanthus inversus syndrome (BPES)**. Many of these represent malformations with significant genetic contributions and may coexist with systemic syndromes **(**Table 4 and 5**)** **[**31].

**Table 4:** Categories of Other Genetic Disorders and Examples.

Disorder Category	Gene examples	Inheritance	OMIM Number
***Malformations***			
**Anophthalmia / Microphthalmia**	SOX2, OTX2 (over 30 human genes associated plus environmental factors)	AD, AR and X-linked	300485
**Lenz Microphthalmia**	BCOR	XLD	300485
**Other**	ALDH1A3, BMP4, RAX, SIX6, SOX2, STRA6, VSX2		
**Cryptophthalmos / Fraser**	FRAS1, FREM2, GRIP1	AR	219000

**Table 5:** Categories of Other Genetic Disorders and Examples.

Disorder Category	Gene examples	Inheritance	OMIM Number
***Ptosis***			
**Blepharophimosis, Ptosis & Epicanthus inversus (BPES)**	FOXL2	AD	110100
**Other Ptosis Diagnoses**	AGRN, ALG14, ALG2, C10orf2, CHAT, CHRNA1, CHRNB1, CHRND, CHRNE, COLQ, DNM2, DOK7, DPAGT1, GFPT1,KIF21A, LRP4, MTM1, MUSK, POLG, POLG2, PREPL, RAPSN, RRM2B, RYR1, SLC25A4, TUBB3		
**Noonan**	PTPN11, RAF1, SOS1, KRAS, BRAF, HRAS, CBL, SHOC2, MAP2K2, MAP2K1, SPRED1	AD	163950
**Kearns-Sayre Syndrome**	Mitochondrial DNA deletion	Mitochondrial	530000
**Myotonic Dystrophy**	DM, DM2	AD (with anticipation)	163950

### Visual impairment in craniofacial disorders

Despite striking craniofacial deformities, structural causes of visual impairment are relatively rare. In a CHOP review of oculoplastic and craniofacial cases, only **~10%** of visual deficits were structural, whereas **90%** were due to **amblyopia**—a preventable and treatable cause of vision loss. Early ophthalmologic assessment and amblyopia management are therefore essential components of multidisciplinary care [32].

## Summary

Embryodysgenesis of the lids and orbit primarily manifests as **malformations** occurring during the **4th–8th weeks of gestation**. The principal categories include **premature craniosynostoses** and **craniofacial clefting disorders**, with a smaller subset of other rare anomalies. Understanding embryogenic mechanisms is crucial for both diagnosis and surgical planning. A multidisciplinary approach—encompassing genetics, pediatrics, ophthalmology, and reconstructive surgery—is indispensable.

Although craniofacial malformations may appear severe, **most vision loss is preventable** through early detection and treatment of amblyopia. Psychosocial support remains equally important to address the aesthetic and emotional challenges faced by affected children and their families.
